# Characterization of the cytochrome P450 monooxygenase genes (P450ome) from the carotenogenic yeast *Xanthophyllomyces dendrorhous*

**DOI:** 10.1186/s12864-017-3942-9

**Published:** 2017-07-19

**Authors:** Pamela Córdova, Ana-María Gonzalez, David R. Nelson, María-Soledad Gutiérrez, Marcelo Baeza, Víctor Cifuentes, Jennifer Alcaíno

**Affiliations:** 1 Departamento de Ciencias Ecológicas y Centro de Biotecnología, Facultad de Ciencias, Universidad de Chile, Las Palmeras 3425, Casilla 653, Santiago, Chile; 2 Department of Microbiology, Immunology and Biochemistry, University of Tennessee Health Science Center, Memphis, TN 38163 USA

**Keywords:** Cytochrome P450, *Xanthophyllomyces dendrorhous*, Astaxanthin synthase

## Abstract

**Background:**

The cytochromes P450 (P450s) are a large superfamily of heme-containing monooxygenases involved in the oxidative metabolism of an enormous diversity of substrates. These enzymes require electrons for their activity, and the electrons are supplied by NAD(P)H through a P450 electron donor system, which is generally a cytochrome P450 reductase (CPR). The yeast *Xanthophyllomyces dendrorhous* has evolved an exclusive P450-CPR system that specializes in the synthesis of astaxanthin, a carotenoid with commercial potential. For this reason, the aim of this work was to identify and characterize other potential P450 genes in the genome of this yeast using a bioinformatic approach.

**Results:**

Thirteen potential P450-encoding genes were identified, and the analysis of their deduced proteins allowed them to be classified in ten different families: CYP51, CYP61, CYP5139 (with three members), CYP549A, CYP5491, CYP5492 (with two members), CYP5493, CYP53, CYP5494 and CYP5495. Structural analyses of the *X. dendrorhous* P450 proteins showed that all of them have a predicted transmembrane region at their N-terminus and have the conserved domains characteristic of the P450s, including the heme-binding region (FxxGxRxCxG); the PER domain, with the characteristic signature for fungi (PxRW); the ExxR motif in the K-helix region and the oxygen-binding domain (OBD) (AGxDTT); also, the characteristic secondary structure elements of all the P450 proteins were identified. The possible functions of these P450s include primary, secondary and xenobiotic metabolism reactions such as sterol biosynthesis, carotenoid synthesis and aromatic compound degradation.

**Conclusions:**

The carotenogenic yeast *X. dendrorhous* has thirteen P450-encoding genes having potential functions in primary, secondary and xenobiotic metabolism reactions, including some genes of great interest for fatty acid hydroxylation and aromatic compound degradation. These findings established a basis for future studies about the role of P450s in the carotenogenic yeast *X. dendrorhous* and their potential biotechnological applications.

**Electronic supplementary material:**

The online version of this article (doi:10.1186/s12864-017-3942-9) contains supplementary material, which is available to authorized users.

## Background

Cytochrome P450 enzymes (P450s or CYPs) constitute a large superfamily of heme containing monooxygenases widely distributed in different organisms from all the domains of life, including animals, plants, fungi and prokaryotes [1]. These enzymes catalyze regio- and stereospecific conversions involved in the oxidative metabolism of a wide range of exogenous and endogenous substrates [2]. They are involved in the biosynthesis of many physiologically important compounds such as sterols, steroid hormones, fatty acids and vitamins [3]. Additionally, they participate in the biosynthesis of a vast array of secondary metabolites in plants, insects and fungi [4], providing them with adaptive advantages for the colonization of specific ecological and/or nutritional niches [5]. Moreover, these enzymes are involved in the detoxification of many xenobiotics such as drugs, pesticides, carcinogens and environmentally contaminating chemicals [3, 6]. The typical reaction catalyzed by these enzymes is: RH + O_2_ + 2e^−^ + 2H^+^ → ROH + H_2_O, where the electrons are transferred from NAD(P)H to P450s by an electron donor [7].

P450 monooxygenase systems have been categorized into 10 classes depending on the proteins participating in the electron transfer [8]. Enzymes representatives of classes II, VIII and IX have been identified in Fungi [9], with class II being the most common class of P450s found in eukaryotic organisms.

Class II eukaryotic P450s are located in the endoplasmic reticulum, and the electron donor is usually the diflavin cytochrome P450 reductase (CPR), which contains FAD and FMN. The electron flow passes from NADPH to FAD to FMN and finally to P450, giving the specificity of the reaction [10, 11]. In most organisms, only one CPR-encoding gene exists, indicating that CPR can reduce many different P450s in a single species. Accordingly, Lah and co-workers [12], through an extensive in silico analysis of several fungal genomes, were able to identify between 20 and 155 putative P450-encoding genes among the analyzed filamentous fungi. However, the number of P450-encoding genes found in yeasts in general was lower, ranging from 2 in *Schizosaccharomyces pombe* to 19 in *Candida albicans*. It has been proposed that a high number of P450s is related to a filamentous growth form in the context of occupying diverse ecological niches [12].

Currently, more than 19,000 P450s have been included in P450 databases and almost 8000 have been named according to the standard P450 nomenclature; this number is continually growing as new genome sequences become available [5, 13, 14]. One of the main reasons for the huge interest in identifying P450s genes from different organisms is their participation in the synthesis of secondary metabolites of biomedical, agricultural and industrial interest [15]. Along these lines, a P450 system has been found to be involved in the last step of the carotenogenesis pathway in the yeast *Xanthophyllomyces dendrorhous*, leading to astaxanthin production [16–18]. Unlike other astaxanthin-producing organisms, *X. dendrorhous* has a single astaxanthin synthase (CrtS, encoded by the *crtS* gene), which is related to a 3A subfamily member cytochrome P450 in vertebrates [16, 17] and to the CYP64 clan in fungi, and catalyzes the hydroxylation and ketolation that convert beta-carotene to astaxanthin. This point suggests that in this yeast only, a unique P450 system has developed and specialized in the synthesis of astaxanthin, as *X. dendrorhous* is the only known organism to date that can synthesize astaxanthin from beta-carotene through a P450 system. Furthermore, the *crtR* gene from *X. dendrorhous*, which encodes a CPR-type enzyme (CrtR), was characterized, and it was proved to be essential for the synthesis of astaxanthin [18]. Additionally, Ukibe and co-workers stated that “*X. dendrorhous CrtS is a unique cytochrome P450 protein that has high specificity for its own P450 reductase”*, as in metabolically engineered *S. cerevisiae* strains with the *X. dendrorhous* carotenogenic genes, astaxanthin production, even in low amounts, was only achieved when CrtS was co-expressed with CrtR [19]. This point suggests that CrtS and CrtR have evolved together, specializing in astaxanthin formation. In addition to astaxanthin synthase, two other cytochrome P450-encoding genes from *X. dendrorhous* have been functionally described: *CYP61* [20] and *CYP51* [21], both involved in ergosterol biosynthesis.

It must to be considered that sedentary organisms, such as fungi and plants, depend on their overall metabolism to meet their environmental conditions and that they have developed complex biochemistries to defend themselves; cytochrome P450 enzymes have proven to play a key role in such processes [12, 22]. Indeed, a protective role against oxidative stress has already been attributed to astaxanthin biosynthesis in *X. dendrorhous* (in which a cytochrome P450 is involved); thus, it is conceivable to expect that this yeast has developed other defense mechanisms that could also include cytochrome P450s (for example, xenobiotics degradation or the synthesis of secondary metabolites besides astaxanthin). The great biotechnological impact of *X. dendrorhous* and the uniqueness of the CrtS-CrtR cytochrome P450 system make this yeast an attractive model to investigate alternative cytochrome P450 redox systems. Taking this into account along with the unquestionable importance of these systems in the metabolism of all organisms and the paucity of knowledge about them in *X. dendrorhous,* this work aimed to provide a detailed genome-wide structural, genetic and phylogenetic characterization of the P450ome of this yeast.

## Methods

### Strain and growth conditions

The wild-type UCD 67–385 (ATCC 24230) *X. dendrorhous* strain was used for genome and transcriptome sequencing. The yeast was grown at 22 °C with constant agitation in YM rich medium (1% glucose, 0.3% yeast extract, 0.3% malt extract and 0.5% peptone). Alternatively, it was grown in minimal medium (MM_V_) supplemented with 2% glucose [23] or 2% succinate [24] as the carbon source.

### Nucleic acid extraction, genome and transcriptome sequencing

DNA was extracted from protoplasts according to [25], and a yield of chromosomal DNA greater than 50 kb was obtained.

Total RNA extraction was performed according to a modified protocol [26] of Chomczynski and Sacchi [27]. Briefly, total RNA was extracted from cell pellets broken through mechanical rupture with 0.5 mm glass beads (BioSpec, Bartlesville, OK, USA) and shaking in a vortex apparatus for 10 min, followed by the addition of Tri-Reagent (AmbionTM, Thermo Fisher Scientific Inc., Waltham, MA, USA). The lysate was incubated for 10 min at room temperature, and 150 μl of chloroform per ml of Tri-Reagent used was added, mixed, centrifuged for 5 min at 4000 x g, after which the aqueous phase was recovered. Then, two consecutive extractions with acidic phenol:chloroform (1:1) were done, and the RNA was precipitated by incubation at room temperature for 10 min with two volumes of isopropanol. The RNA was washed with 75% ethanol, suspended in RNase-free H_2_O and quantified spectrophotometrically at 260 nm in a V-630 UV-Vis Spectrophotometer from JASCO (JASCO, Easton, MD, USA), according to Sambrook et al. [28].

Genomic and transcriptomic sequencing was conducted as described previously [29]. For transcriptomic analyses, total RNA was extracted from yeast cultures at the early exponential (~18 h) and initial stationary (~72 h) phases of growth, grown in minimal media supplemented either with glucose or succinate as the sole carbon source, generating a total of four different conditions (G18, G72, S18 and S72: **G**lucose or **S**uccinate at **18** or **72** h of culture) representing two types of metabolism in *X. dendrorhous* (fermentative in glucose and aerobic in succinate) to allow a better analysis of gene expression in different conditions. The assembly and analysis of the obtained transcriptomes were performed using the CLC Genomics Workbench 5 program.

### Identification and characterization of the putative P450-encoding genes

The whole collection of contigs and scaffolds of the genome and transcriptomes was analyzed with the CLC Genomic Workbench software and compared by BLAST analysis against two P450 specialized databases: the David Nelson cytochrome P450 homepage web site (http://drnelson.uthsc.edu/CytochromeP450.html) [13] and the fungal cytochrome P450 database (http://p450.riceblast.snu.ac.kr) [5]. In this way, the putative *X. dendrorhous* P450-encoding genes were identified. Gene sequences were analyzed with Geneious 10.0.2, and the analyses of the deduced protein sequences were performed using programs available online: Protein Calculator v3.3 (available at http://protcalc.sourceforge.net) was used to estimate protein parameters such as molecular weight and pI; TMpred (available at http://www.ch.embnet.org/software/TMPRED_form.html) [30] was used to predict transmembrane regions; the CYP450 Engineering database (available at https://cyped.biocatnet.de/) and the programs JPred (available at www.compbio.dundee.ac.uk/jpred/) [31] and SWISS-MODEL (available at https://swissmodel.expasy.org/) [32, 33] were used to predict secondary structure elements characteristic of P450 enzymes (the protein prediction servers were visited for the last time on March 28, 2017).

To search for cis-regulatory elements, the promoter region, defined as the 1500 nucleotides immediately upstream of the translation start codon of each P450-encoding gene of *X. dendrorhous,* was analyzed with four programs: MATCH [34], PATCH (both available at http://gene-regulation.com/pub/programs.html), TFBIND (available at http://tfbind.hgc.jp/) [35] and JASPAR (available at http://jaspar.genereg.net/) [36]. The programs MATCH, PATCH and TFBIND use the TRANSFAC database, while JASPAR uses the JASPAR database (the cis-regulatory element websites were visited for the last time on March 28, 2017).

Phylogenetic analysis was carried out using Clustal Omega (available at http://www.ebi.ac.uk/Tools/msa/clustalo) and Simple Phylogeny (available at http://www.ebi.ac.uk/Tools/phylogeny/simple_phylogeny) for protein multiple alignments and phylogenetic tree construction, respectively (the alignment and phylogenetic tree construction web sites were visited for the last time on March 28, 2017). For phylogenetic tree construction, the neighbor-joining method was used, and the obtained tree was visualized with the software MEGA 6.0 [37].

The sequences of P450s from other organisms used in the construction of the phylogenetic tree were the following: *S. cerevisiae*: *ERG11* [NP_011871.1], *ERG5* [P54781.1]; *Aspergillus nidulans*: *bzuA* [AAL10516.1], *ppoC* [AAT36614.1], *ppoA* [AAR88626.1], *ahbB* [AAR15377.1], *stcF* [AAC49196.1], *stcB* [AAC49196.1], *phacA* [CAB43093.1], *phacB* [ABB20530.1], *ivoC* [CBF77085.1], *CYP62* [XP_681093.1]; *Aspergillus nonius*: *cypA* [AAS90045.1]; *Aspergillus fumigatus*: *CYP51a* [ACF17705.1], *CYP51b* [AAK73660.1], *gliC* [EDP49542.1], *gliF* [AAW03300.1], *ftmE* [BAH23999.1], *ftmC* [BAH23996.1], ftmG [BAH24001.1]; *Aspergillus clavatus*: *CYP619C2* [ACG60892.1], *CYP619C3* [ACG60891.1]; *Aspergillus oryzae*: *nicA* [BAC01275.1]; *Aspergillus niger*: *bph* [P17549.1]; *Aspergillus parasiticus*: *aflU* [Q6UEH4.1], *aflQ* [AAS66031.1]; *Cryptococcus neoformans*: *ERG11* [AAF35366.1]; *Aspergillus flavus*: *CYP51Ap* [EED56341.1], *CYP51Bp* [EED50354.1], *AF115* [AAT65721.1]; *Phanerochaete chrysosporium*: *CYP51* [ACI23621.1]; *Coprinopsis cinereus*: *eln2* [BAA33717.1]; *Fusarium graminearum*: *tri1* [AAQ02672.1], *tri11* [BAC22120.1], *tri4* [AAK53584.1]; *Fusarium sporotrichioides*: *tri4* [AAB72032.1]; *Fusarium oxysporum*: *fum6* [ACB12553.1], *CYP505* [BAA82526.1], *CYP55* [P23295.]; *Myrothecium roridum*: *tri4* [AAC49958.1]; Leptosphaeria maculans: *sirB* [AAS92544.1], *sirC* [AAS92547.1], *sirE* [AAS92549.1]; *Penicillium paxilli*: *paxP* [AAK11528.1], *paxQ* [AAK11527.1]; *Candida albicans*: *dit2* [CAK54651.1], *alk8* [CAA75058.1], *CYP51* [XP_716761]; *Candida maltosa*: *P450alk* [CAA39367.1], *CYP52A3-b* [AAC60531.1]; *Candida glabrata*: *CYP61* [KTB21909.1]; *Chaetomium chiversii*: *radP* [ACM42407.1]; *Streptomyces venezuelae*: *piKC* [AAC68886.1]; *Nectria haematococca*: *PDAT9* [AAC01762.1]; *Trichosporon cutaneum*: *P450nor* [BAB60855.1]; *Gibberella intermedia*: *P450–4* [Q701P2.1], *P450–1* [CAF31353.1]; *Gibberella moniliformis*: *fum2* [AAN74815.2]; *Heterobasidion irregulare*: *CYP56* [XP_009550156], *CYP65* [ETW74855.1], *CYP59* [ETW81982.1], *CYP60* [ETW77818.1], *CYP62* [XP_009548132.1], *CYP68* [ETW81986.1]; *Yarrowia lipolytica*: *ALK3* [BAA31435.1]; *ALK8* [BAA31440]; *ALK5* [BAA31437.1]. The gene names are indicated, followed by the P450 protein sequence accession numbers (GenBank or NCBI reference sequence).

## Results and discussion

### Identification and structural analysis of *X. dendrorhous* P450-encoding genes

Regarding the P450 genes, the cytochrome P450 homepage website built by Dr. David Nelson has been available since 1995 [13], and in addition, a fungal specialized cytochrome P450 database has been available online since 2008 [5]. The assembled genome (available in our laboratory and also published by Sharma et al. (2015) [38]) and transcriptomes of *X. dendrorhous* were compared against these two databases with the BLAST tool, allowing us to identify several putative P450 genes. As a result, 13 potential cytochrome P450-encoding genes, including *crtS*, *CYP51* and *CYP61*, were found in the *X. dendrorhous* genome and were uploaded to GenBank (Table 1). Notably, the *X. dendrorhous* P450ome would be one of the largest found in yeasts, since previous studies have shown that most yeasts (unicellular fungi) have fewer than ten cytochrome P450s, with a few exceptions such as *Candida albicans* (19 cytochrome P450s) and *Yarrowia lipolytica* (17 cytochrome P450s), unlike filamentous fungi that have often higher numbers of P450s encoding genes [12, 22]. Accordingly, the 13 cytochrome P450s of *X. dendrorhous* were named following the prescriptions of the International P450 Nomenclature Committee, where they fell into 10 families, each containing only one member except for CYP5139 (with three members) and CYP5492 (with two members) (Table 1).

**Table 1:** *X. dendrorhous* P450 encoding genes

Gene					Protein			Best Nelson database BLAST Hit				Best FCPD database BLAST Hit			
Name	GenBank ID	***N°***: Exons (bp)	***N°***: Introns (bp)	ORF (bp)	Length (aa)	kDa	pI	Name	specie	% id	e-value	Name	specie	% id	e-value
*CYP5139Q1*	[EU713462.1]	***18***: 118–49–117-92-215-34-143-60-100-97-111-34-72-88-58-74-25-187	***17***: 82–115–72-123-83-89-73-82-76-77-76-90-148-63-76-77-90	1674	557	62.63	5.93	CYP5139B1	*Cryptococcus neoformans*	35	9e-97	Ba134	*Bjerkandera adusta*	38.06	3e-99
*(crtS* ^*a*^ *)*															
*CYP51F1*	[KY775138]	***10***: 141–70–47-63-60-462-86-233-83-408	***9***: 354–95–79-80-86-110-90-87-90	1653	550	61.78	6.89	CYP51F1	*Cryptococcus neoformans*	62	0	Sl1069	*Serpula lacrymans*	61.84	0
*(CYP51* ^*a*^ *)*															
*CYP61A1*	[KY775139]	***9***: 156–152–114-75-81-441-169-320-73	***8***: 317–82–90-83-84-79-116-111	1581	526	59.60	6.95	CYP61A1	*Cryptococcus neoformans*	63	0	Tm002	*Tremella mesenterica*	64.74	0
*(CYP61* ^*a*^ *)*															
*CYP5139P1*	[KY775128]	***18***: 139–49–117-92-224-34-137-60-112-97-111-34-69-88-61-74-25-190	***17***: 119–150–160-119-88-126-168-123-192-120-228-118-104-85-136-91-95	1713	570	63.65	7.16	CYP5139B1	*Cryptococcus neoformans*	40	e-110	Sl7053	*Serpula lacrymans*	44.72	1e-118
*CYP5490A1*	[KY775129]	***16***: 96–115–143-90-148-146-250-13-26-4-94-97-114-76-47-170	***15***: 243–92–111–89-91–84-99-77-74-60-87-129-94-114-73	1629	542	61.71	7.61	CYP5141A4	*Phanerochaete chrysosporium*	37	1e-85	Ha085	*Heterobasidion irregulare*	37.86	4e-89
*CYP5491A1*	[KY775130]	***12***: 260–125–38-132-72-165-82-28-16-193-211-193	***11***: 113–75–87-96-85-104-78-94-88-129-109	1515	504	56.42	8,61	CYP5230A1	*Puccinia graminis*	26	1e-38	Sl7039	*Serpula lacrymans*	25.88	2e-46
*CYP5492A1*	[KY775131]	***14***: 205–72–69-24-14-95-97-147-273-160-66-178-103-186	***13***: 82–78–93-76-86-86-91-74-81-104-111-99-94	1689	562	64.72	7.52	CYP5230A1	*Puccinia graminis*	29	4e-51	Sl7039	*Serpula lacrymans*	30.12	4e-67
*CYP5493A1*	[KY775132]	***14***: 176–200–38-177-171-71-94-85-28-16-130-66-260-201	***13***: 130–74–92-75-117-81-74-67-77-72-80-78-86	1713	570	63.31	6.95	CYP5272B1	*Neurospora discreta*	26	3e-42	Sl7039	*Serpula lacrymans*	28	1e-53
*CYP53C10*	[KY775133]	***14***: 63–148–97-103-290-60-64-154-146-206-72-18-82-93	***13***: 84–107–146-110-96-101–88-98-87-83-100-114-111	1596	531	59.12	7.20	CYP53C2	*Phanerochaete chrysosporium*	54	e-154	Ba130	*Bjerkandera adusta*	56.17	1e-154
*CYP5494A1*	**[**KY775134]	***14***: 357–67–77-6-126-109-17-241-56-142-52-69-163-291	***13***: 108–104–85-69-119-106-79-106-83-75-81-105-139	1773	590	67.77	6.28	CYP63A2	*Phanerochaete chrysosporium*	34	2e-78	Sl7144	*Serpula lacrymans*	35.82	3e-85
*CYP5495A1*	[KY775135]	***6***: 388–311–244-156-323-177	***5***: 100–120–86-120-95	1599	532	59.65	6.93	CYP5134B1P	*Neosartorya fischeri*	23	6e-25	Mlp016	*Melampsora larici-populina*	27.29	2e-27
*CYP5492A2*	[KY775136]	***14***: 208–72–69-24-14-95-97-147-291-160-66-214-103-168	***13***: 109–71–102-82-119-101-124-87-111-106-115-135-155	1728	575	65.61	7.28	CYP5134A1	*Fusarium oxysporum*	27	8e-50	Sl7039	*Serpula lacrymans*	31.86	2e-68
*CYP5139R1*	[KY775137]	***19***: 182–76–41-74-18-20-78-103-137-196-80-98-34-160-58-102-24-87-82	***18***: 97–69–107-90-86-91-103-85-84-108-106-93-103-80-108-74-93-77	1650	549	61.97	7.47	CYP5139B2	*Cryptococcus neoformans*	41	e-118	Tm008	*Tremella mesenterica*	41.81	1e-120

^a^The names *crtS, CYP61* and *CYP51* were given to these genes when they were previously described by [17, 20] and [21], respectively. The total number of exons (third column) and of introns (fourth column) are in bold and italics

The sequence analyses of the genomic and cDNA versions of each P450-encoding gene allowed us to determine their exon-intron structure and deduce the encoded polypeptide (Table 1). The structure of each gene was defined by sequence alignments, which were manually edited. The *X. dendrorhous* P450 genes have between 6 and 19 exons, ranging in size from as small as 4 bp to 462 bp. The intronic sequences ranged from 72 to 354 bp having the canonical GT-AG donor and acceptor splice site at their extremes; except for the fourth intron of *CYP5490A1* and the first intron of *CYP5492A2*, which had the less common GC-AG splice sites. Additionally, the GT-CA splice sites were observed in the eleventh intron of *CYP5139R1*, which was confirmed by Sanger sequencing in several *X. dendrorhous* strains.

### Transcriptomic analysis of P450 gene expression

To obtain more information about the P450 genes of *X. dendrorhous*, four transcriptomes of the wild-type strain UCD 67–385 were analyzed, and the level of each of the P450 gene transcripts was estimated by calculating its RPKM (reads per kilobase per million mapped reads) value, which mirrors the molar concentration of the transcript in the starting sample normalized to the length of the RNA and the total number of reads [39]. The analyzed transcriptomes were obtained from yeast cultures at the early exponential (~18 h) and initial stationary (~72 h) phases of growth, grown in minimal media supplemented either with glucose or succinate as the sole carbon source, generating a total of four different conditions (G18, G72, S18 and S72: **G**lucose or **S**uccinate at **18** or **72** h of culture) (Table 2). In general, the results show that the P450-encoding gene transcripts have similar RPKM values in the four studied conditions, but changes are observed in some cases such as the *crtS*, *CYP5491A1*, *CYP5492A1*, *CYP5493A1* and *CYP5494A1* genes, whose RPKM values in the G72 condition are higher than the values at the G18 condition, suggesting induction at late growth phases. This has also been observed in previous studies for the *crtS* gene [24].

**Table 2:** RPKM values of the *X. dendrorhous* P450-encoding gene transcripts

	Condition^a^				Normalization by G18		
Gene	G18	G72	S18	S72	G72/G18	S18/G18	S72/G18
*crtS*	85.4	596.3	87.9	245.2	7.0	1.0	2.9
*CYP51*	51.7	48.8	40.9	39.2	0.9	0.8	0.8
*CYP61*	110.9	133.0	51.7	41.7	1.2	0.5	0.4
*CYP5139P1*	27.4	57.0	64.0	67.4	2.1	2.3	2.5
*CYP5490A1*	20.1	23.4	19.0	44.8	1.2	0.9	2.2
*CYP5491A1*	7.8	31.7	5.4	11.4	4.1	0.7	1.5
*CYP5492A1*	4.6	180.2	11.8	31.1	39.4	2.6	6.8
*CYP5493A1*	11.4	32.9	7.8	16.9	2.9	0.7	1.5
*CYP53C10*	7.0	8.6	7.7	6.3	1.2	1.1	0.9
*CYP5494A1*	9.3	25.2	30.1	29.5	2.7	3.3	3.2
*CYP5495A1*	19.1	43.9	25.9	27.5	2.3	1.4	1.4
*CYP5492A2*	5.8	5.4	16.1	25.1	0.9	2.8	4.3
*CYP5139R1*	44.7	49.0	19.2	28.2	1.1	0.4	0.6

^a^The four conditions G18, G72, S18 and S72 were previously described in the text

On other hand, when the RPKM values of the S18 and S72 conditions were compared, no major changes were observed. Additionally, it was observed that the *CYP5494A1* and *CYP5492A2* transcript RPKM values were higher in the S18 condition than in G18, suggesting differential expression depending on the carbon source or possibly related to the type of metabolism performed, either fermentative for growth on glucose or aerobic for succinate. As mentioned above, most of the P450 transcripts did not show major changes; this may be because some of the studied genes encode proteins involved in essential functions, such as *CYP51* and *CYP61*. On the other hand, it is possible that some of the studied P450 genes are induced by an external factor; this may be the reason why no changes were observed in the studied conditions. An example of the latter occurs in *Aspergillus niger*, in which the cytochrome P450 benzoate-para-hydroxylase gene is only induced when benzoate is added to the culture medium [40]. A similar phenomenon was observed in the yeast *Candida tropicalis*, which can metabolize phenol as a carbon source using a P450 system, where the cytochrome P450 content was induced by phenol [41].

It should be noted that among P450s, Cyp51 (lanosterol 14 alpha-demethylase) is the only widely conserved enzyme, having orthologs in organisms from bacteria to humans; this status suggests that the enzyme could represent the common ancestor of all P450s [42]. In addition, Cyp51 is the only enzyme in the P450 family that fulfills the same function in all biological kingdoms, namely, three-step sterol 14-demethylation in sterol biosynthesis [43]. In mammals, the biosynthesis of cholesterol is regulated by the SREBP (sterol regulatory element binding protein) pathway, which promotes the transcription of genes such as *CYP51* in response to low cholesterol levels in the cell. This pathway has recently been characterized in the yeast *S. pombe*, and it was shown that the two P450 genes in this organism, *CYP61* and *CYP51*, which are involved in ergosterol biosynthesis, are under the regulation of the SREBP pathway [44]. SREBP (named Sre1 in fungi) binds to SRE (sterol regulatory element) sequences at the promoter region of the target genes. To determine whether this transcriptional factor could be regulating P450-encoding genes in *X. dendrorhous*, the promoter region, comprising 1500 nucleotides upstream of the translation start codon of each gene, was bioinformatically analyzed. The SRE sequences identified by at least two of the programs used in the analyses are detailed in Table 3. According to the criteria used, it was possible to identify potential SRE sequences in most sequences except for those from the *crtS*, *CYP5491A1* and *CYP5494A1* genes, identifying possible SRE sites in the promoter region of P450 genes involved in ergosterol biosynthesis (*CYP51* and *CYP61*) consistent with previous works. This result suggests that the majority of the P450s encoding genes from *X. dendrorhous* could be regulated, at least in part, by the same regulatory pathway as their potential common ancestor *CYP51*.

**Table 3:** SRE sequences detected in the promoter regions of *X. dendrorhous* P450-encoding genes

Gene	Strand	Sequence (5′-3′)	Position (start-end)		Program
*CYP51*	+	GTGGGGTCAC	−685	−694	JASPAR/TFBIND
	+	ATCACCTCTC	−95	−104	JASPAR/TFBIND
*CYP61*	+	ATCAACTGAC	−1412	−1421	JASPAR/TFBIND
	+	ATCACCAGAG	−519	−528	JASPAR/TFBIND
*CYP5139P1*	+	CTCACACCAC	−1048	−1057	JASPAR/TFBIND
	+	ATCACATGAT	−262	−271	JASPAR/TFBIND
*CYP5490A1*	+	GTTGGGTGAG	−709	−718	JASPAR/TFBIND
*CYP5492A1*	+	GTCATCTGAT	−1267	−1276	JASPAR/TFBIND
	−	ATCACCCCAA	−367	−376	JASPAR/PATCH
	+	ATCACGTCA	−314	−323	JASPAR/TFBIND
*CYP5493A1*	+	ATCAACCCAC	−413	−422	JASPAR/TFBIND/PATCH
*CYP53C10*	+	GTGACATGAT	−369	−378	JASPAR/TFBIND
*CYP5495A1*	+	GTGGGCTGAA	−1057	−1066	JASPAR/TFBIND
	+	ATCACGTGAA	−102	−111	JASPAR/TFBIND/PATCH/MATCH
*CYP5492A2*	−	ATCACCTCAC	−985	−994	JASPAR/PATCH
	−	ATCAAGCCAG	−522	−531	JASPAR/PATCH
*CYP5139R1*	+	GAGAGGTGAT	−313	−322	JASPAR/TFBIND

### Analysis of deduced amino acid sequences of P450s

The 13 potential cytochrome P450 gene sequences of *X. dendrorhous* were analyzed to find the corresponding ORFs, and the encoded proteins were deduced. The ORFs varied in length from 1296 to 1773 bp. The sizes of the deduced proteins ranged from 504 to 590 amino acids, consistent with the sizes of previously described P450 proteins [45, 46]. The predicted molecular masses varied from 56.2 to 67.77 kDa, and the isoelectric points (pIs) ranged from 5.93 to 8.61 (Table 1).

Regarding the subcellular localization of the deduced P450 proteins, a putative hydrophobic transmembrane segment at the amino terminus was predicted by TMpred [30] analyses in all the deduced amino acid sequences. This finding is important as this feature allows class II P450 enzymes to anchor to the endoplasmic reticulum membrane [9].

Many studies have demonstrated that P450s share little sequence similarity, except for a few conserved domains needed to preserve their tertiary structure and enzymatic function [22]. The most conserved motif is the heme-binding region FxxGxRxCxG (also known as the CxG motif) containing the axial Cys ligand that binds to the heme. The motifs ExxR and PER form the E-R-R triad, which is important for locking the structure of the heme pocket in place and ensuring the stabilization of the core structure. Moreover, ExxR and FxxGxRxCxG contain the three most conserved amino acids in the cytochrome P450 protein family: the glutamic acid and arginine in the ExxR motif and the heme-binding cysteine in the CxG motif [47]. The last conserved motif, AGxDTT, contributes to oxygen binding and activation [22, 46].

**Fig. 1:**
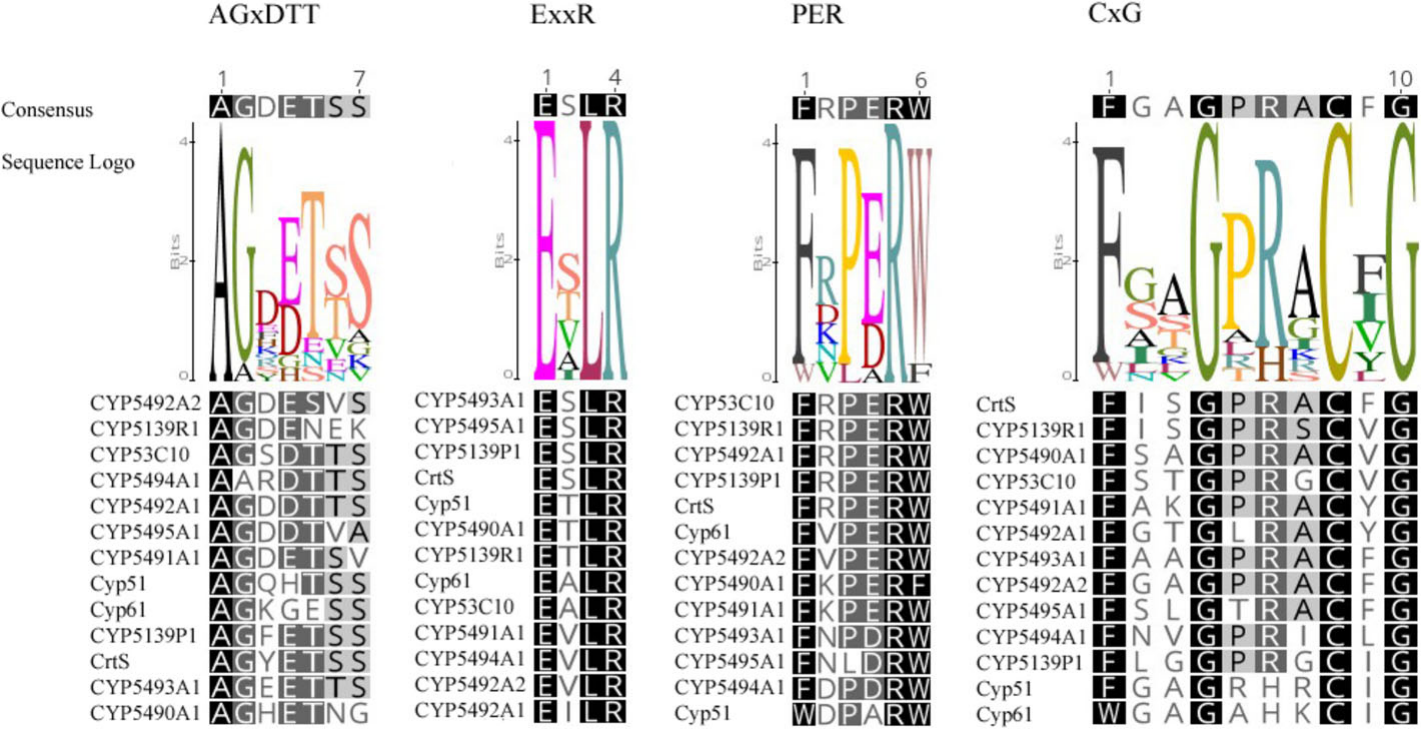
Conserved motifs in the P450 proteins of *X. dendrorhous.* Multiple alignments of the conserved motifs AGxDTT, ExxR, PER and CxG of *X. dendrorhous.* Alignments are highlighted by percentage of identity as follows: *black*: 100% identity, *gray*: >80% identity, *light gray*: >60% identity and *white*: <60% identity. Sequence logos show the conservation and relative frequency of each amino acid within each conserved motif. Alignments and sequence logos were constructed using the Geneious software

The ExxR and FxxGxRxCxG motifs are present in the 13 predicted P450s from *X. dendrorhous*, and all of them have the three conserved amino acids (Fig. 1). Syed and Mashele performed an extensive comparative analysis of the P450 signature motifs among the fungal kingdom [47], and our results are in agreement with this previous work. Among the *X. dendrorhous* ExxR motifs of P450s, the ESLR pattern was the most abundant (in 4 sequences), followed by ETLR and EVLR (in 3 sequences each), EALR (in 2 sequences) and EILR (in 1 sequence). Interesting, between the glutamic acid and the arginine at the first and last position of this motif, respectively, all the *X. dendrorhous* P450s contain a leucine at the third position, which has also been shown to be the most predominant residue at this position in the fungal P450s. Serine, threonine and valine were shown to be the most frequent residues at the second position of the *X. dendrorhous* P450s ExxR motif, in agreement with what has been previously reported [47]. Each *X. dendrorhous* P450 showed a different FxxGxRxCxG motif pattern; however, all of them contained the conserved cysteine at the eighth position and had glycine at the fourth and tenth position. All the sequences had a phenylalanine at the first position, with the exception of Cyp61, which has a tryptophan. This last observation is not surprising since it has been shown that tryptophan is the second most frequent residue at this position among 47 fungal P450 sequences analyzed [22].

It has been reported that arginine is the predominant residue at the sixth position of the FxxGxRxCxG motif, and this is the case in all of the *X. dendrorhous* P450s except for Cyp51 and Cyp61, which have a histidine. In previous studies, this residue was shown to be the most common at this position in the Cyp61 family [47], the second most frequent among 47 fungal P450s analyzed and the most frequent among 105 bacterial P450s analyzed [22]. For the CxG pattern (the three last residues of the FxxGxRxCxG motif), four *X. dendrorhous* P450s have the CFG pattern, three each have a CIG and a CVG pattern, two have a CYG pattern and one has a CLG pattern. These results are different from what have been reported for this motif, as it was previously found that CPG was the most predominate triad in basidiomycetes, followed by CIG, CLG and CVG in second, third and fourth place, respectively. The most frequent pattern in *X. dendrorhous* (CFG) only represented 0.21% of a total of 4304 fungal P450 sequences and approximately 0.14% of 2859 Basidiomycota P450 sequences analyzed previously [47]. Six of the 13 P450 proteins identified in *X. dendrorhous* (including CrtS), have an aromatic aminoacid (F or Y) in the middle of the CxG triad, both uncommon residues at this position among fungal P450s (among the 4304 fungal P450 sequences previously analyzed, 0.21% and 0.09% were CFG and CYG, respectively) [47]. Although the effect of the different amino acids in the middle position of the triad CxG is still unknown, previous studies have demonstrated that different P450 families show preference for determined residues at this position suggesting that amino acids in this motif play a role in determining P450 structure, activity and substrate specificity [47]. Considering that the CrtS enzyme, which has the CFG triad, has the unique ability to convert β-carotene to astaxanthin by finally introducing both, a ketone and a hydroxyl group at both β-ionone rings of β-carotene [17], it is possible that the predominant CFG triad found in the P450s of *X. dendrorhous* contributed to a new substrate specificity related to the enzymes functions. Moreover, in a phylogenetic study of the *X. dendrorhous* P450s (see below), those having aromatics amino acids in the middle position of the CxG triad (CYP5491A1, CYP5492A1, CYP5492A2, CYP5493A1 and CYP5495A1) showed to be closely related, suggesting related functions.

The third conserved motif described as a P450 signature: PER [48], with the characteristic signature PxRW for fungi [46], was also found in the 13 *X. dendrorhous* P450s (Fig. 1). Although this motif has four amino acids, the two previous residues were also considered in the analysis, giving a six-amino-acid sequence, as a phenylalanine residue two positions before the conserved proline of the PxRW motif is highly conserved in most P450s [22]. Accordingly, in all but one of the *X. dendrorhous* P450s, a phenylalanine residue was found at the first position. As expected, in the third position, the most common residue was a proline, followed in sequence by a less conserved glutamic acid and a completely conserved arginine at the fifth position. Other than the conserved arginine, the most common amino acid was a tryptophan at the sixth position, consistent with previous descriptions of fungal P450s [22].

The last conserved motif, AGxDTT, also known as the oxygen binding domain (OBD), was found in all of the P450s of *X. dendrorhous*, but it was the least conserved in sequence, with the first (an alanine in all sequences), second (a glycine in most cases) and fifth (a threonine in most cases) positions being the most conserved ones (Fig. 1). At the fourth position, the most common residues were glutamic acid and aspartic acid, in agreement with other fungal P450s described [22, 46].

These results showed that all of the deduced gene products from all of the identified *X. dendrorhous* P450 genes have all the characteristic signature motifs described for this superfamily of proteins.

### P450 structure

**Fig. 2:**
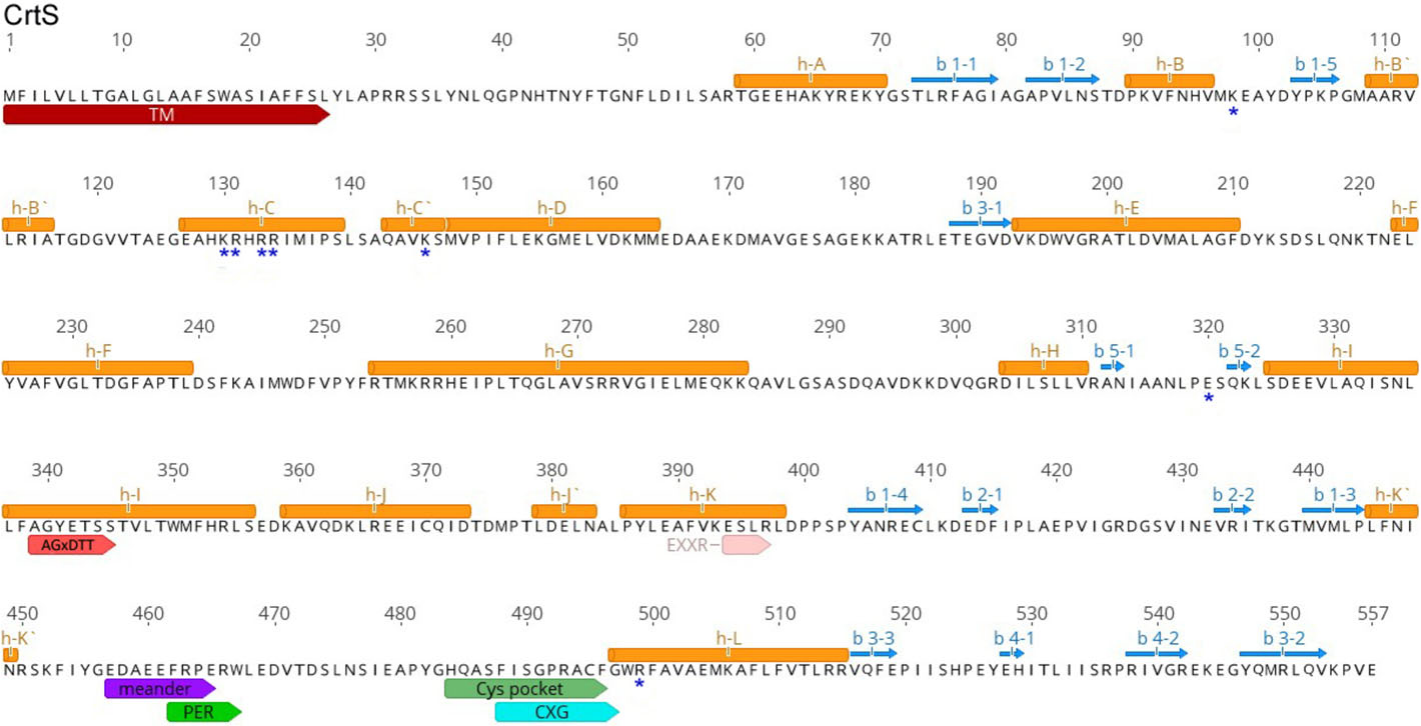
Analysis of CrtS protein from *X. dendrorhous*. Characteristic conserved secondary elements of P450 proteins are shown along the CrtS protein. Alpha helices (h) and beta strands (b) are represented as *orange cylinders* and *blue arrows*, respectively. The transmembrane region (TM) and conserved regions such as the “meander loop” and the “Cys pocket” are also highlighted. The location of the conserved motifs AGxDTT, ExxR, PER and CxG are shown along the protein. Residues involved in the interaction CrtS-CrtR according to [52] are marked with *asterisks*

In the last few years, several studies have analyzed the structure of different P450 proteins. The increasing number of crystal structures published, together with comparative analyses of different P450 structures, have revealed that despite the low sequence identity (10–30%), members of this superfamily of proteins have a conserved structural core [49]. In general, the secondary structure elements of these enzymes include at least four beta sheets (β1 to β4) and 12 alpha helices (αA to αL). Also, in some structures, an additional beta sheet (β5) or insertions forming additional alpha helices (named B′, C′, J’, K′ and K″) have been found, with K′ and J’ being the most frequent ones [50, 51]. Considering the above, the *X. dendrorhous* P450s proteins deduced using the CYP450 Engineering database (https://cyped.biocatnet.de) were analyzed. Using the “Standard Numbering” tool of this P450 structure database, positive results were obtained for proteins Cyp61, Cyp51, CrtS, Cyp5139P1, Cyp5490A1, Cyp53C10, Cyp5494A1 and Cyp5139R1 of *X. dendrorhous*; among them, the deduced secondary structures of the first two have been previously reported [20, 21]. The SWISS-MODEL [32, 33] and JPred [31] servers available online were used to predict the secondary structure elements in the rest of the *X. dendrorhous* P450s (Cyp5491A1, Cyp5492A1, Cyp5493A1, Cyp5495A1 and Cyp5492A2). In this way, the characteristic regions known as the “Cys pocket” and “the meander loop” in P450 enzymes were identified in all of the deduced P450s of *X. dendrorhous*. The most conserved secondary structural elements (αA-αL helices and β1- β4 sheets) according to [51] were all found in the predicted P450 proteins (Fig. 2 and Additional file 1). In Fig. 2, the predicted secondary structural elements of CrtS are shown as an example.

A less conserved structural element was β5, which was not identified in four of the 13 P450s analyzed. Furthermore, the B′ and C′ alpha helices, which are not described as highly conserved in previous studies [50, 51], were present in ten of the *X. dendrorhous* P450s. In contrast, alpha helices J’ and K′ were found in all the predicted P450 proteins. Thus, the predicted secondary structure elements indicate that the proteins identified in this work have the characteristic secondary structural elements of P450 enzymes.

As previously mentioned, P450 enzymes require an electron donor partner. In *X. dendrorhous*, the CrtR protein, a CPR enzyme, plays that role and has been studied principally for its involvement in the last step of astaxanthin biosynthesis together with the CrtS enzyme (P450). Regarding this, a previous study [52] predicted which residues were important for the interfacial interaction between CrtS and CrtR. In the CrtS protein, eight residues were identified as implicated in the formation of salt bridges with CrtR: K98, K130, R131, R133, R134, K146, E320 and R499. Among them, only K130, R134, K146 and E320 appeared to be specific for the CrtS-CrtR interaction [52]. The sequences of all the *X. dendrorhous* P450s were analyzed to determine whether these eight residues are conserved. In CrtS, K98 is positioned between the helix αB and β strands 1–5, and it is 32 residues away from the next conserved residue, K130, which is positioned in the helix αC. Taking this into account, residues potentially equivalent to CrtS K98 were found in ten of the 13 P450s analyzed (Fig. 2 and Additional file 1), and in the case of Cyp53C10, a histidine (H95) was found in this position, which would replace the function of the conserved arginine since both residues are positively charged amino acids. The next residues in CrtS that would be involved in the CrtS-CrtR interaction are grouped in the helix αC, comprising the region from position 130 to 134 (K130, R131, R133, R134). It must be noted that in this region in CrtS, five positively charged residues are in consecutive positions (although H132 would not be involved in formation of salt bridges as previously reported [52]), followed by K146 at helix αC’, 11 positions later. The region from helix αC to helix αD of all *X. dendrorhous* P450s was screened for the presence of equivalent residues (Additional file 2). In general, at least three positively charged residues were found grouped inside the helix αC except in the proteins Cyp51 and Cyp61, in which only a pair of positive residues were found. A residue potentially equivalent to K146 was found in all but one (Cyp5493A1) of the deduced proteins, replaced by an arginine in some cases (Additional file 2). Although it is difficult to ensure the equivalent correspondence since in some cases there was more than one possibility, residues potentially equivalent to CrtS E320 (positions between αH and αI) were found in all of the *X. dendrorhous* P450s proteins, including an aspartic acid in place of a glutamic acid in two cases. Finally, in the case of R499 from CrtS, which is positioned three residues after the conserved cysteine of the Cys pocket region, potentially equivalent residues were found in ten of the P450s, the arginine being replaced in some cases by a lysine and absent in Cyp51, Cyp61 and Cyp5495A1 (Additional file 1).

In general, the residues potentially involved in the interaction with the cytochrome P450 reductase CrtR are conserved among the *X. dendrorhous* P450s. Nevertheless, previous studies have demonstrated that a *X. dendrorhous crtR* ^−^ mutant is viable [18]; thus, P450s enzymes involved in essential processes such as ergosterol biosynthesis, as in the case of Cyp51 and Cyp61, should be able to interact with other electron donor partners such as the cytochrome b5 reductase/cytochrome b5 pathway [53], and it is possible that some of the salt-bridge-forming residues identified in the CrtS protein are not conserved in other P450s, because less specific interactions are needed compared to the CrtS-CrtR interaction.

### Phylogenetic study

As previously mentioned, the first analyses of the P450 sequences showed that the thirteen P450s from *X. dendrorhous* belong to 10 different protein families: CYP51, CYP61, CYP5139 (with three members), CYP549A, CYP5491, CYP5492 (with two members), CYP5493, CYP53, CYP5494, and CYP5495 (Table 1).

**Fig. 3:**
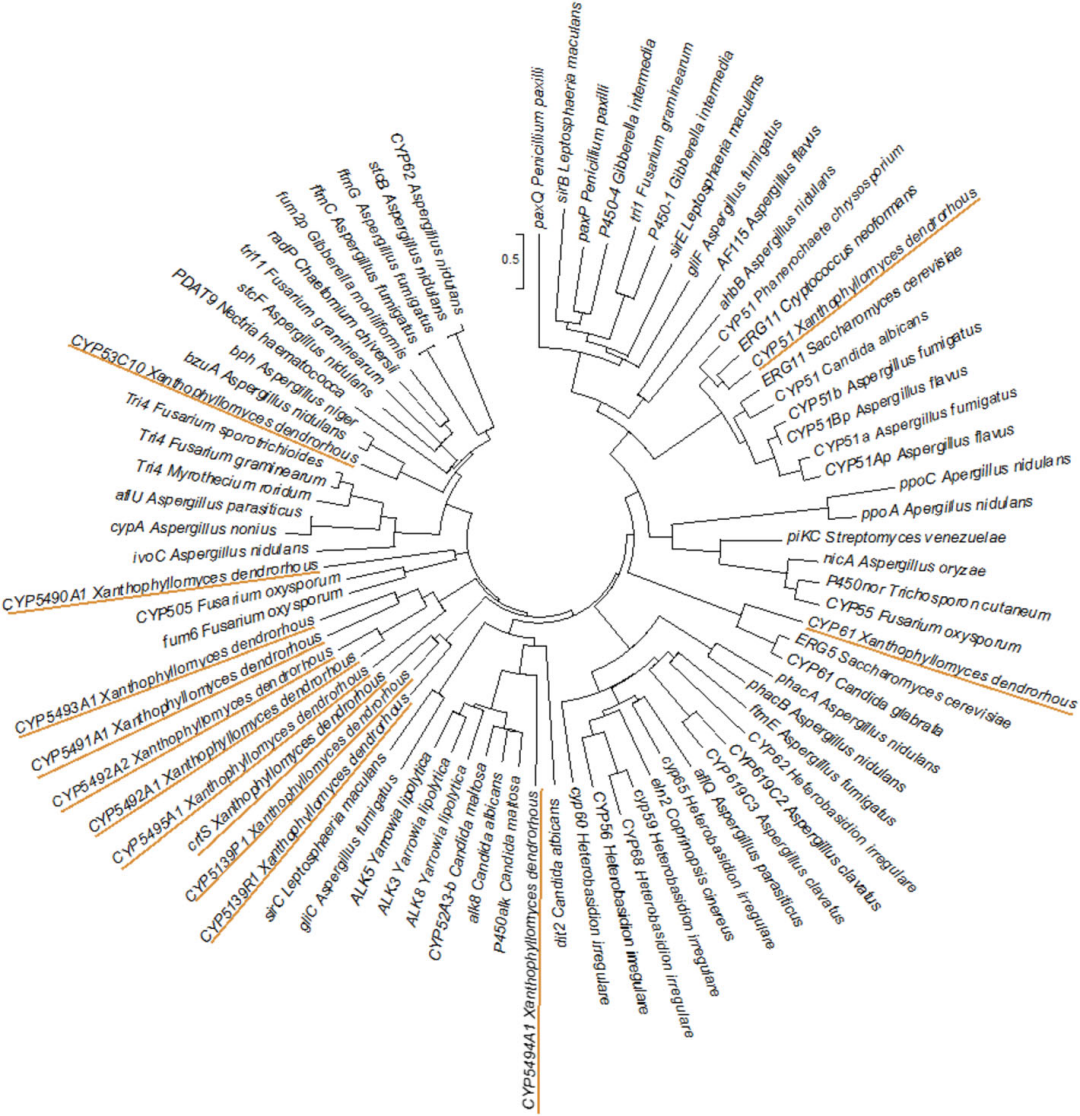
Phylogenetic analysis tree of all *X. dendrorhous* P450s and other fungal P450s*.* The phylogenetic tree was created with the Simple Phylogeny tool using the neighbor-joining method with the amino acid sequences of all the P450s from *X. dendrorhous* (*underlined*) and 67 functionally characterized P450s from other organisms. In the tree, the names of the P450-encoding genes are detailed, followed by the organism of origin. The accession numbers of the sequences are indicated in the Methods section

The functions of the proteins CrtS (carotenoid biosynthesis), Cyp61 and Cyp51 (ergosterol biosynthesis) were described in previous studies [17, 20, 21]. To determine the possible functions of the other ten P450s of *X. dendrorhous*, a phylogenetic tree was constructed using all the *X. dendrorhous* P450 sequences and the amino acid sequences of 67 previously functionally characterized P450 proteins obtained from the Fungal Cytochrome P450 database (http://p450.riceblast.snu.ac.kr/char_p450.php) and other works [9]. Protein multiple alignment was done with the online tool Clustal Omega, and the phylogenetic tree was constructed using the neighbor-joining statistical method with the Simple Phylogeny tool using the default parameters. The tree, originally in the Newick format, was visualized with MEGA 6.0 software [37], and the resulting tree is presented in Fig. 3.

In general, the thirteen P450s of *X. dendrorhous* could be separated into seven groups. In the first two groups, the *X. dendrorhous* Cyp51 and Cyp61 proteins grouped together with described Cyp51 and Cyp61 from other microorganisms, respectively. This was expected as the involvement of both enzymes in sterol biosynthesis has been extensively characterized in different organisms, including *X. dendrorhous* [20, 21]. A third group contained Cyp5494A1 of *X. dendrorhous*, which was grouped apart from the other P450s of this yeast, and whose closest relatives are the “alk” proteins of *Yarrowia lipolytica* and different *Candida* species. This observation is very interesting, because the *ALK* genes from different organisms have been studied due to their importance in the assimilation of *n*-alkanes and fatty acids as well as their participation in xenobiotic metabolism [9, 54]. A fourth group included the CrtS, Cyp5139P1 and Cyp5139R1 proteins of *X. dendrorhous*, which were considerably distant from any other of the characterized fungal P450s. In this group, the CrtS protein has a known function in the astaxanthin biosynthesis. Based on the high identity percentage between Cyp5139P1 and CrtS (47% identity, 65% similitude), it is plausible that Cyp5139P1 could have a related function. Since Cyp5139P1 and Cyp5139R1 (this last one showed 35% identity and 54% similitude with CrtS) did not show similarity with any other functionally characterized enzyme, experimental studies are required to suggest their potential function. A fifth group included Cyp5491A1, Cyp5492A1, Cyp5493A1, Cyp5495A1 and Cyp5492A2 from *X. dendrorhous,* of which Cyp5492A1 and Cyp5492A2 were the most closely related, belonging to the same P450 family. In this case, no functionally characterized P450s from other fungi were related to these proteins, so it was not possible to suggest a potential function for this group of P450s, opening the opportunity to find new, undescribed functions for these proteins. In a sixth group, Cyp5490A1 of *X. dendrorhous* grouped with the proteins encoded by the *fum6* and *CYP505* genes of *Fusarium oxysporum*, which are involved in secondary metabolism reactions regarding the synthesis of the mycotoxin fumonisin and the ω-1 to ω-3 carbon hydroxylation of fatty acids, respectively [9, 55, 56]. Regarding fatty acid hydroxylation, the microbial production of hydroxyl fatty acids is of commercial interest because these compounds are widely used in the chemical, food, and cosmetic industries as starting materials for the synthesis of polymers and as additives for the manufacture of lubricants, emulsifiers, and stabilizers. Additionally, they have antibiotic, anti-inflammatory, and anticancer activities with potential medicinal uses [57]. In this sense, the function of Cyp5490A1 could be of great interest if it is related to the functions of the proteins encoded by *fum6* or *CYP505*. Overall, the results suggest that Cyp5490A1 may participate in secondary metabolism. In the last group, the protein Cyp53C10 of *X. dendrorhous* grouped together with the proteins encoded by the genes *bzuA* and *bph* (benzoate para-hydroxylase) of *Aspergillus nidulans* and *Aspergillus niger*, respectively. The *bzuA* gene *of A. nidulans* encodes a protein orthologous to the benzoate para-hydroxylase-encoding gene *bph* of *A. niger;* in both cases, the encoded enzymes participate in primary and xenobiotic metabolism (benzamide and benzoate degradation) [58, 59]. This may be of great interest as Cyp53C10 of *X. dendrorhous* could be involved in the degradation of aromatic xenobiotic compounds such as benzoate and benzamide.

Finally, the phylogenetic analysis separated the thirteen P450s of *X. dendrorhous* into groups revealing the potential functions of these recently discovered genes, ranging from primary functions, such as sterols biosynthesis, to secondary and xenobiotic metabolism, including reactions of biotechnological interest.

## Conclusions

The present study reveals that the carotenogenic yeast *X. dendrorhous* possesses thirteen P450-encoding genes, whose products may be involved in primary and secondary metabolism reactions. In the case of CrtS, Cyp51 and Cyp61, their functions in carotenogenesis and sterol synthesis, respectively, have been previously described. The possible functions of the other identified P450s include reactions involved in primary and xenobiotic metabolism such as fatty acid hydroxylation and aromatic compound degradation. These findings lay a foundation for future experiments to elucidate the role of the different P450s in *X. dendrorhous* and the possibility of discovering new functions and potential applications for this diverse group of enzymes.

## Additional files

Additional file 1: Figure S1. Analysis of secondary structure elements in P450 proteins from *X. dendrorhous*. Characteristics conserved secondary elements of P450 proteins are shown along the thirteen P450 proteins of *X. dendrorhous*. Alpha helices (h) and beta-strands (b) are represented as orange cylinders and blue arrows, respectively. The transmembrane region (TM) and conserved regions such as the “meander loop” and the “Cys pocket” are also highlighted. The locations of the conserved motifs AGxDTT, ExxR, PER and CxG are shown along the proteins. Residues potentially involved in the interaction between P450 and CrtR are indicated in red. (JPEG 1040 kb)

Additional file 2: Figure S2. Multiple alignment of the region from helix αC to helix αD of all *X. dendrorhous* P450s. An alignment was made using Geneious v.10 software, and residues were colored by polarity. Asterisks indicate the most conserved positively charged residues. (JPEG 344 kb)

## Abbreviations

CPR: Cytochrome P450 reductase
FAD: Flavin adenine dinucleotide
FMN: Flavin mononucleotide
MMv: Vogel minimum medium
NADPH: Nicotinamide adenine dinucleotide phosphate
OBD: Oxygen-binding domain
ORF: Open reading frame
P450 or CYP: Cytochrome P450
pI: Isoelectric point
RPKM: Reads per kilobase per million mapped reads
