# Supplementary figures and images for: Characterization of the cytochrome P450 monooxygenase genes (P450ome) from the carotenogenic yeast Xanthophyllomyces dendrorhous
Source: BMC Genomics. 2017 Jul 19;18:540. doi: 10.1186/s12864-017-3942-9 (PMC5516332; doi:10.1186/s12864-017-3942-9)

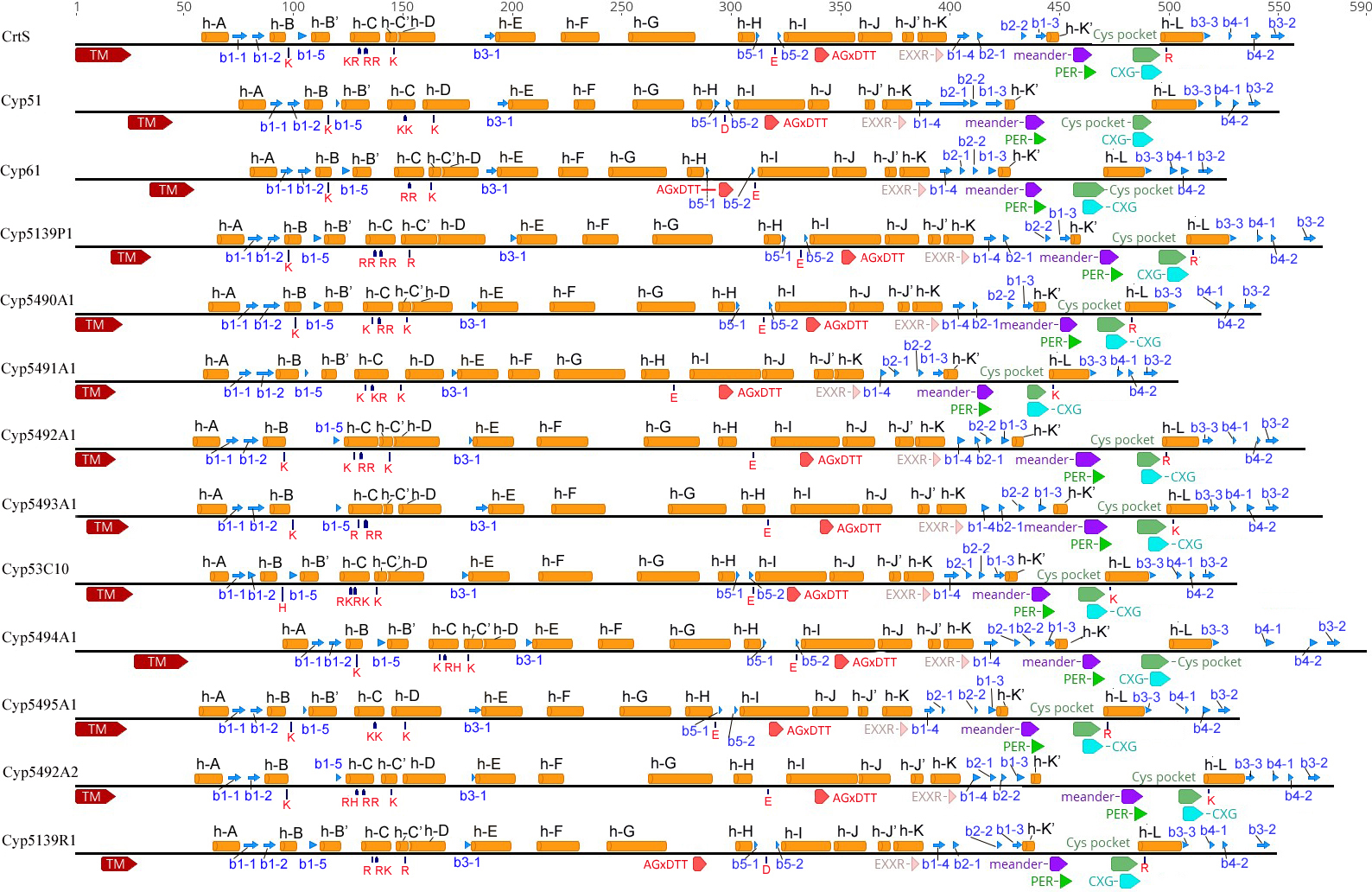

Supplement: Supplementary file 1 — Analysis of secondary structure elements in P450 proteins from X. dendrorhous. Characteristics conserved secondary elements of P450 proteins are shown along the thirteen P450 proteins of X. dendrorhous. Alpha helices (h) and beta-strands (b) are represented as orange cylinders and blue arrows, respectively. The transmembrane region (TM) and conserved regions such as the “meander loop” and the “Cys pocket” are also highlighted. The locations of the conserved motifs AGxDTT, ExxR, PER and CxG are shown along the proteins. Residues potentially involved in the interaction between P450 and CrtR are indicated in red. (JPEG 1040 kb) [file 12864_2017_3942_MOESM1_ESM.jpg]

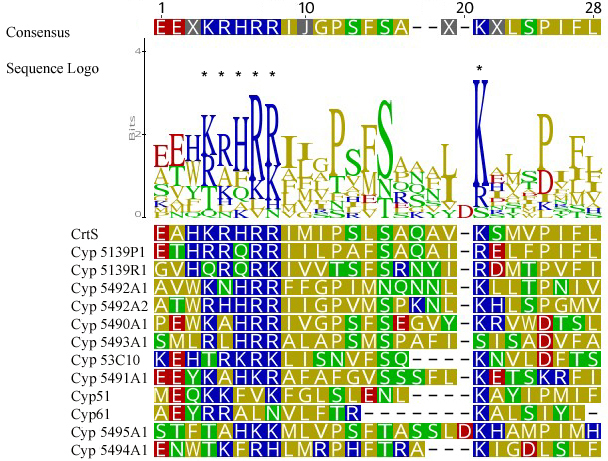

Supplement: Supplementary file 2 — Multiple alignment of the region from helix αC to helix αD of all X. dendrorhous P450s. An alignment was made using Geneious v.10 software, and residues were colored by polarity. Asterisks indicate the most conserved positively charged residues. (JPEG 344 kb) [file 12864_2017_3942_MOESM2_ESM.jpg]
